# Supplementary material for: Giant spin ensembles in waveguide magnonics
Source: Nat Commun. 2022 Dec 8;13:7580. doi: 10.1038/s41467-022-35174-9 (PMC9732049; doi:10.1038/s41467-022-35174-9)
Supplement: Supplementary file 1 — Supplementary Information [file 41467_2022_35174_MOESM1_ESM.pdf]

Supplementary Material for "Giant spin ensemble in waveguide magnonics"

Zi-Qi Wang,[1] Yi-Pu Wang,[1,*] Jiguang Yao,[1] Rui-Chang Shen,[1]
Wei-Jiang Wu,[1] Jie Qian,[1] Jie Li,[1] Shi-Yao Zhu,[1] and J. Q. You[1,†]

[1]*Interdisciplinary Center of Quantum Information,
State Key Laboratory of Modern Optical Instrumentation,
and Zhejiang Province Key Laboratory of Quantum Technology and Device,
Department of Physics, Zhejiang University, Hangzhou 310027, China*

(Dated: November 8, 2022)

I. Experimental methods

A. Device design

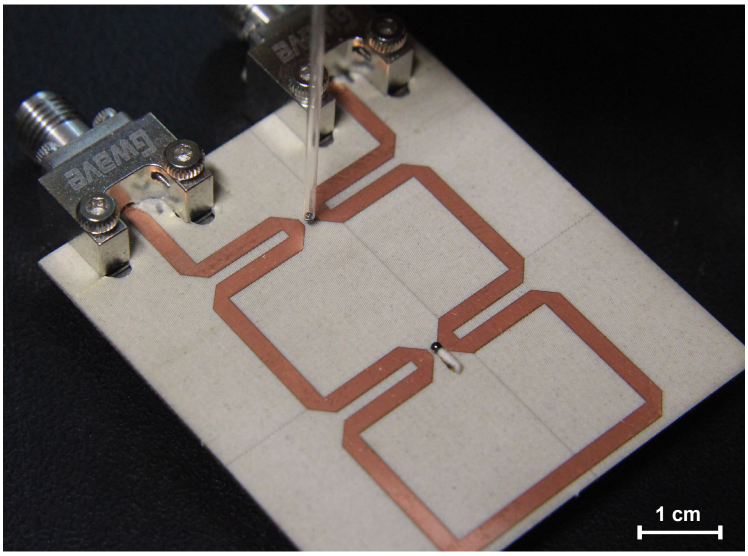

Supplementary Fig. 1 **The photograph of our device.**

B. Tuning magnon mode frequency

The magnon mode frequency can be tuned in a large range by adjusting the bias magnetic field. As shown in Fig. 2a, the magnon mode frequency is monitored using a loop antenna placed above the YIG sphere. The antenna is made by winding the center conductor of a semi-rigid coaxial cable. From the measured reflection mapping depicted in Fig. 1**e** of the main text, it can be seen that the linewidth of the Kittel mode hardly varies over the measured frequency range.

To observe the collective behaviour of the nested two GSEs, it is necessary to individually tune one of the spin ensembles. For the homogeneously magnetized YIG sphere, the magnon mode frequency is given by $\omega_{\rm m} = \gamma\,(H_{\rm e} + H_{\rm A})$, where $H_{\rm e}$ is the bias magnetic field and $H_{\rm A}$ is the anisotropy field. The anisotropy field contains the contributions from the shape anisotropy field (SAF) $H_{\rm S}$ and magnetocrystalline anisotropy field (MAF) $H_{\rm M}$. For a spherical sample, the SAF does not change the frequency of the magnon mode when rotating the sphere. Therefore, we neglect the SAF in the following calculation of the magnon mode frequency for a YIG sphere. In contrast, the MAF makes the frequency of the magnon mode dependent on the orientation of the YIG sphere with respect to the bias field. Thus, we can utilize the MAF to tune the frequency of the magnon mode by rotating the sphere. Here, effective demagnetization factor is used to represent the magnetocrystalline anisotropy field,

$$H_{\rm an} = -\overset{\leftrightarrow}{N}_{\rm an}\, M_0, \qquad \text{(S1)}$$

where $\overleftrightarrow{N}_{\rm an}$ is the tensor of the effective demagnetization factor and M_0 is the magnetization. The eigenfrequency can be determined by projecting the demagnetization factor onto the Cartesian coordinate axes, which is expressed as

$$\omega_0 = \left[(\gamma N_{11} M_0 + \gamma H_{\rm e0} - \gamma N_{33} M_0) (\gamma N_{22} M_0 + \gamma H_{\rm e0} - \gamma N_{33} M_0) - \gamma^2 N_{12}^2 M_0^2 \right]^{1/2}, \tag{S2}$$

where γ is the gyromagnetic ratio and N_{ij} is the projection of the demagnetization factor in Cartesian coordinates. Also, the z-axis coincides with the direction of M_0. For our spherical samples of the YIG crystal, which has a cubic lattice, the components of $\overleftrightarrow{N}_{\rm an}$ can be expressed as [1]

$$\begin{aligned} N_{11}^{\rm an} &= -3 \frac{H_{\rm A}}{M_0} \sin^2\theta_{\rm H} \sin^2 2\phi_0, \\ N_{22}^{\rm an} &= -3 \frac{H_{\rm A}}{M_0} \sin^2\theta_{\rm H} \left(1 - \frac{1}{4}\sin^2 2\phi_0\right), \\ N_{12}^{\rm an} &= -3 \frac{H_{\rm A}}{M_0} \sin^2\theta_{\rm H} \cos\theta_{\rm H} \sin 4\phi_0, \\ N_{33}^{\rm an} &= \frac{H_{\rm A}}{M_0} \left(1 + \cos^2 2\theta_{\rm H} - \sin^4\theta_{\rm H} \sin^2 2\phi_0\right). \end{aligned} \tag{S3}$$

The orientations of the coordinate axes are depicted in Fig. 2, where ϕ_0 and $\theta_{\rm H}$ are indicated. Substituting Eq. (S3) into Eq. (S2), we obtain the resonance frequency

$$\begin{aligned} \frac{\omega_0^2}{\gamma^2} = & \left\{ H_{\rm e0} + H_{\rm A} \times \left[\frac{3}{2} + \frac{1}{2}\cos 4\theta_{\rm H} + \left(-\frac{15}{8} + 2\cos 2\theta_{\rm H} - \frac{1}{8}\cos 4\theta_{\rm H} \right) \sin^2 2\phi_0 \right] \right\} \\ & \times \left\{ H_{\rm e0} + H_{\rm A} \left[2\cos 4\theta_{\rm H} + \left(\frac{1}{2}\cos 2\theta_{\rm H} - \frac{1}{2}\cos 4\theta_{\rm H} \right) \sin^2 2\phi_0 \right] \right\} - \frac{9}{4} H_{\rm A}^2 \sin^2\theta_{\rm H} \sin^2 2\theta_{\rm H} \sin^2 4\phi_0. \end{aligned} \tag{S4}$$

In our experiment, the magnetization of the YIG sphere is confined to the {110} plane. We only change $\theta_{\rm H}$, leaving $\phi_0 = \frac{\pi}{4}$ unaltered. Considering that the anisotropic field is much smaller than the bias magnetic field ($H_{\rm A} \ll H_{\rm e0}$), we have

$$\frac{\omega_0}{\gamma} = H_{\rm e0} + H_{\rm A} \left(-\frac{3}{16} + \frac{5}{4}\cos 2\theta_{\rm H} + \frac{15}{16}\cos 4\theta_{\rm H} \right). \tag{S5}$$

In the main text, the angle dependence of the resonance frequency derived from Eq. (S5) is plotted in Fig. 1**h**, which is consistent with the experimental result.

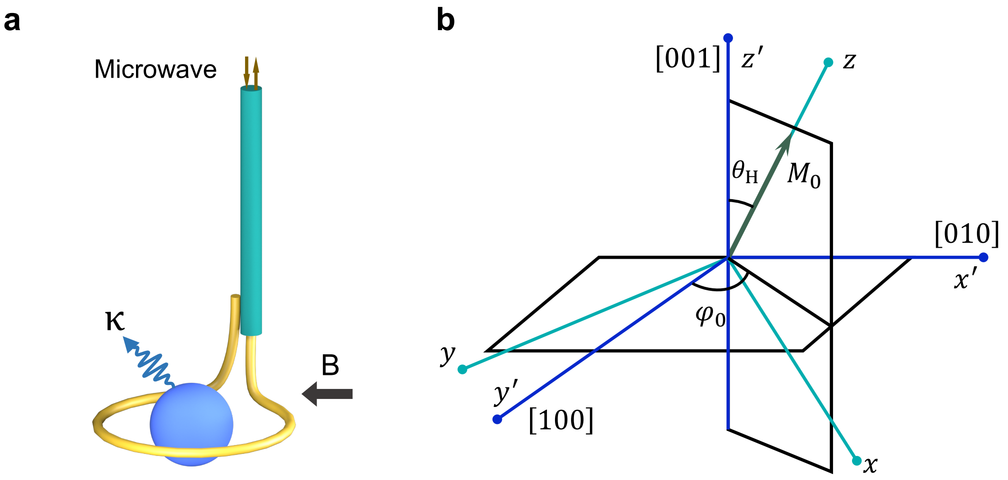

Supplementary Fig. 2 **Characterization of the magnon mode in the YIG sphere.** **a**. Schematic of the experimental setup for measuring the reflection spectrum. The loop antenna is made by winding the center conductor of a semi-rigid coaxial cable. **b**. Schematic of the crystal axes and magnetization of a YIG crystal. The blue axes represent hard axes [001], [010], and [100] of the YIG crystal. The bias magnetic field is applied along the z direction, and the cyan axes represent the magnetization-referenced coordinate axes.

II. Single giant spin ensemble

As illustrated in Fig. 3, the magnon mode in a YIG sphere interacts with the rightwards ($\hat{p}_{\rm k}$) and leftwards ($\hat{q}_{\rm k}$) travelling photon modes. Using the Holstein-Primakoff transformation [2] under low-lying excitations, we can write the Hamiltonian of the system as

$$\begin{aligned} H_1/\hbar =& \tilde{\omega}_{\rm m}\hat{a}_{\rm m}^\dagger\hat{a}_{\rm m} + \sum_{\rm k}\omega_{\rm k}\hat{p}_{\rm k}^\dagger\hat{p}_{\rm k} + \sum_{\rm k}\omega_{\rm k}\hat{q}_{\rm k}^\dagger\hat{q}_{\rm k} \\ &+ \sum_{\rm k} g_1\left(\hat{a}_{\rm m}+\hat{a}_{\rm m}^\dagger\right)\left(\hat{p}_{\rm k}^\dagger+\hat{q}_{\rm k}^\dagger+\hat{p}_{\rm k}+\hat{q}_{\rm k}\right) + \sum_{\rm k} g_2\left(\hat{a}_{\rm m}+\hat{a}_{\rm m}^\dagger\right)\left[e^{-i\varphi}\left(\hat{p}_{\rm k}^\dagger+\hat{q}_{\rm k}\right)+e^{i\varphi}\left(\hat{p}_k+\hat{q}_k^\dagger\right)\right], \end{aligned} \tag{S6}$$

where $\hat{a}_{\rm m}^\dagger$ ($\hat{a}_{\rm m}$) is the creation (annihilation) operator of the magnon mode, and $\tilde{\omega}_{\rm m} = \omega_{\rm m} - i\beta$ is its complex frequency, with the real and imaginary parts representing the resonant frequency and intrinsic (non-radiative) decay rate of the magnon mode, respectively. The creation (annihilation) operators of the rightward and leftward travelling photon modes are $\hat{p}_{\rm k}^\dagger$ ($\hat{p}_{\rm k}$) and $\hat{q}_{\rm k}^\dagger$ ($\hat{q}_{\rm k}$), respectively, which follow the commutation relations $[\hat{p}_{\rm k}, \hat{p}_{\rm k'}] = \delta(k-k')$ and $[\hat{q}_{\rm k}, \hat{q}_{\rm k'}] = \delta(k-k')$. The last row in Eq. (S6) represents the interaction between the travelling photons and the giant spin ensemble (GSE). Here the GSE is effectively achieved by coupling the YIG sphere to the waveguide at two separated points, with the coupling strengths g_1 and g_2, respectively. In our work, identical coupling strengths between the magnon mode and travelling photon modes are considered in both rightward and leftward propagating directions.

The travelling phase $\varphi = \omega_{\rm m}t_{12} = \omega_{\rm m}\left(S_{\rm m}^2 - S_{\rm m}^1\right)/v$ is introduced, where v and t_{12} are the microwave speed in the waveguide and the travelling delay time between the two coupling points. The effective size of the GSE is $L_{\rm m} = S_{\rm m}^2 - S_{\rm m}^1$. From Eq. (S6), we obtain the Heisenberg equation for $\hat{a}_{\rm m}$, $\hat{q}_{\rm k}$, and $\hat{p}_{\rm k}$, as well as their conjugate operators:

$$\frac{d\hat{a}_{\rm m}}{dt} = -i\tilde{\omega}_{\rm m}\hat{a}_{\rm m}(t) - ig_1\sum_{\rm k}\left(\hat{p}_{\rm k}^\dagger+\hat{p}_{\rm k}+\hat{q}_{\rm k}^\dagger+\hat{q}_{\rm k}\right) - ig_2\sum_{\rm k}\left[e^{-i\varphi}\left(\hat{p}_{\rm k}^\dagger+\hat{q}_{\rm k}\right)+e^{i\varphi}\left(\hat{p}_{\rm k}+\hat{q}_{\rm k}^\dagger\right)\right], \tag{S7}$$

$$\frac{d\hat{a}_{\rm m}^\dagger}{dt} = i\tilde{\omega}_{\rm m}\hat{a}_{\rm m}^\dagger(t) + ig_1\sum_{\rm k}\left(\hat{p}_{\rm k}^\dagger+\hat{p}_{\rm k}+\hat{q}_{\rm k}^\dagger+\hat{q}_{\rm k}\right) + ig_2\sum_{\rm k}\left[e^{-i\varphi}\left(\hat{p}_{\rm k}^\dagger+\hat{q}_{\rm k}\right)+e^{i\varphi}\left(\hat{p}_{\rm k}+\hat{q}_{\rm k}^\dagger\right)\right], \tag{S8}$$

$$\frac{d\hat{p}_{\rm k}}{dt} = -i\omega_{\rm k}\hat{p}_{\rm k} - ig_1\left[\hat{a}_{\rm m}(t)+\hat{a}_{\rm m}^\dagger(t)\right] - ig_2e^{-i\varphi}\left[\hat{a}_{\rm m}(t)+\hat{a}_{\rm m}^\dagger(t)\right], \tag{S9}$$

$$\frac{d\hat{p}_{\rm k}^\dagger}{dt} = i\omega_{\rm k}\hat{p}_{\rm k}^\dagger + ig_1\left[\hat{a}_{\rm m}(t)+\hat{a}_{\rm m}^\dagger(t)\right] + ig_2e^{-i\varphi}\left[\hat{a}_{\rm m}(t)+\hat{a}_{\rm m}^\dagger(t)\right], \tag{S10}$$

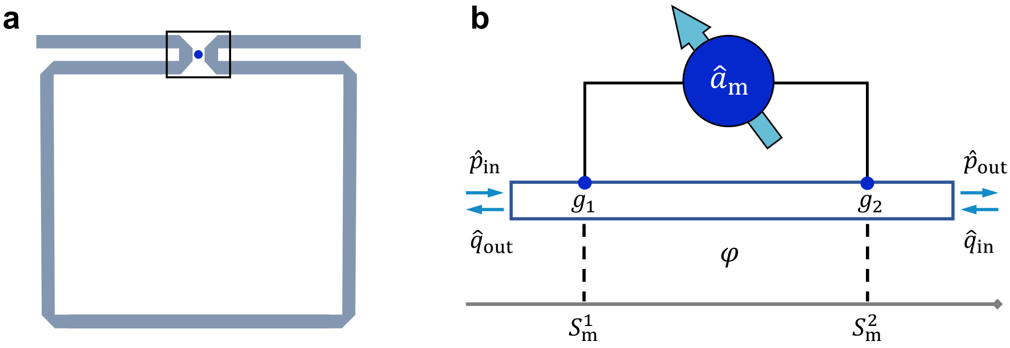

Supplementary Fig. 3 **Single giant spin ensemble. a**. Schematic of the giant spin ensemble (GSE), where a YIG sphere interacts with the meandering waveguide (gray) at two coupling points and the rectangular frame depicts the GSE. **b**. Details of the GSE-waveguide coupled system.

$$\frac{d\hat{q}_{\rm k}}{dt}=-i\omega_{\rm k}\hat{q}_{\rm k}-ig_1\left[\hat{a}_{\rm m}(t)+\hat{a}_{\rm m}^{\dagger}(t)\right]-ig_2e^{i\varphi}\left[\hat{a}_{\rm m}(t)+\hat{a}_{\rm m}^{\dagger}(t)\right], \tag{S11}$$

$$\frac{d\hat{q}_{\rm k}^{\dagger}}{dt}=i\omega_{\rm k}\hat{q}_{\rm k}^{\dagger}+ig_1\left[\hat{a}_{\rm m}(t)+\hat{a}_{\rm m}^{\dagger}(t)\right]+ig_2e^{i\varphi}\left[\hat{a}_{\rm m}(t)+\hat{a}_{\rm m}^{\dagger}(t)\right], \tag{S12}$$

with both $\hat{p}_{\rm k}$ and $\hat{q}_{\rm k}$ including all allowed k. We utilize the density of states $J(\omega)$ to convert the sum of all k into an integral. Then, we have

$$\begin{aligned}\int_0^{\infty}\hat{p}_{\rm k}(t)J(\omega)d\omega=&\int_0^{\infty}e^{-i\omega(t-t_{\rm i})}\hat{p}_{\rm k}\left(t_{\rm i}\right)J(\omega)d\omega-ig_1\int_0^{\infty}J(\omega)d\omega\int_{t_{\rm i}}^{t}d\tau e^{-i\omega(t-\tau)}\left[\hat{a}_{\rm m}(\tau)+\hat{a}_{\rm m}^{\dagger}(\tau)\right]\\&-ig_2e^{-i\varphi}\int_0^{\infty}J(\omega)d\omega\int_{t_{\rm i}}^{t}d\tau e^{-i\omega(t-\tau)}\left[\hat{a}_{\rm m}(\tau)+\hat{a}_{\rm m}^{\dagger}(\tau)\right],\end{aligned} \tag{S13}$$

$$\begin{aligned}\int_0^{\infty}\hat{p}_{\rm k}(t)J(\omega)d\omega=&\int_0^{\infty}e^{-i\omega(t-t_{\rm f})}\hat{p}_{\rm k}\left(t_{\rm f}\right)J(\omega)d\omega+ig_1\int_0^{\infty}J(\omega)d\omega\int_{t_{\rm f}}^{t}d\tau e^{-i\omega(t-\tau)}\left[\hat{a}_{\rm m}(\tau)+\hat{a}_{\rm m}^{\dagger}(\tau)\right]\\&+ig_2e^{-i\varphi}\int_0^{\infty}J(\omega)d\omega\int_{t_{\rm f}}^{t}d\tau e^{-i\omega(t-\tau)}\left[\hat{a}_{\rm m}(\tau)+\hat{a}_{\rm m}^{\dagger}(\tau)\right],\end{aligned} \tag{S14}$$

where Eqs. (S13) and (S14) are the solutions at initial ($t<t_{\rm i}$) and final ($t>t_{\rm f}$) conditions, respectively. In order to obtain the input-output relation and the effective Hamiltonian of the GSE, we assume $t_{\rm i}=0$ to simplify the integral and then we get

$$\sqrt{\kappa_1}\hat{p}_{\rm out}=\sqrt{\kappa_1}\hat{p}_{\rm in}-\left(ig_1^2+ig_2g_1e^{-i\varphi}\right){\rm Re}[{\rm I}\left({\rm t},\tau\right)], \tag{S15}$$

where

$$\hat{p}_{\rm in}=\frac{1}{\sqrt{2\pi}}\int_0^{\infty}e^{-i\omega(t-t_{\rm i})}\hat{p}_{\rm k}\left(t_{\rm i}\right)J(\omega)d\omega, \tag{S16}$$

$$\hat{p}_{\rm out}=\frac{1}{\sqrt{2\pi}}\int_0^{\infty}e^{-i\omega(t-t_{\rm f})}\hat{p}_{\rm k}\left(t_{\rm f}\right)J(\omega)d\omega, \tag{S17}$$

$$I\left(t,\tau\right)=\int_0^{\infty}J(\omega)d\omega\int_0^{t_{\rm f}}d\tau e^{i\omega(t-\tau)}\left(\hat{a}_{\rm m}^{\dagger}\left(\tau\right)+\hat{a}_{\rm m}\left(\tau\right)\right), \tag{S18}$$

and

$$\int_0^{\infty}e^{-ikx}dx=\pi\delta\left(x\right)-i{\rm PV}\left(\frac{1}{k}\right). \tag{S19}$$

The dispersive term related to the Cauchy principal value (PV) gives a specific value after truncation, leading to the Lamb shift in the energy level [3]. In the GSE, the interference between two coupling points allows its Lamb shift to be determined by its eigenfrequency. By substituting the integral into Eq. (S7) and under the rotating-wave approximation, we obtain the equation for the steady-state average $\langle\hat{a}_{\rm m}\rangle$:

$$-i\left(\omega_{\rm m}+\sqrt{\kappa_1\kappa_2}\sin\varphi\right)\langle\hat{a}_{\rm m}\rangle-\left(\kappa_1+\kappa_2+2\sqrt{\kappa_1\kappa_2}\cos\varphi+\beta\right)\langle\hat{a}_{\rm m}\rangle-i\left(\sqrt{\kappa_1}+\sqrt{\kappa_2}e^{i\varphi}\right)\hat{p}_{\rm in}=0, \tag{S20}$$

where $\kappa_{1(2)}=2\pi g_{1(2)}^2$ are the radiative decay rates of the GSE at the two coupling points. In our work, we always place the YIG sphere at the center of two mitered corners of the waveguide to ensure that the radiative decay rates at the two coupling points are the same ($\kappa_1=\kappa_2=\kappa$), and S_{21} can be obtained by substituting Eq. (S20) into Eq. (S15),

$$S_{21}(\omega)=\frac{\hat{p}_{\rm out}}{\hat{p}_{\rm in}}=1+\frac{\kappa_{\rm G}}{i\left(\omega-\omega_{\rm m}-\kappa\sin\varphi\right)-\kappa_{\rm T}}, \tag{S21}$$

where $\kappa_{\rm T}=\kappa_{\rm G}+\beta$ is the total dissipation rate of the GSE, and $\kappa_{\rm G}$ is the radiative decay rate of the GSE, which is given by

$$\kappa_{\rm G}=2\kappa\left(1+\cos\varphi\right). \tag{S22}$$

III. Nested two giant spin ensembles

The Hamiltonian of the nested two GSEs interacting with a meandering waveguide can be written as

$$\begin{aligned} H_2/\hbar = & \tilde{\omega}_{\mathrm{i}}\hat{a}_{\mathrm{i}}^{\dagger}\hat{a}_{\mathrm{i}} + \tilde{\omega}_{\mathrm{o}}\hat{a}_{\mathrm{o}}^{\dagger}\hat{a}_{\mathrm{o}} + \sum_{\mathrm{k}}\omega_{\mathrm{k}}\hat{p}_{\mathrm{k}}^{\dagger}\hat{p}_{\mathrm{k}} + \sum_{\mathrm{k}}\omega_{\mathrm{k}}\hat{q}_{\mathrm{k}}^{\dagger}\hat{q}_{\mathrm{k}} \\ & + \sum_{\mathrm{k}} g_{\mathrm{o}}\left(\hat{a}_{\mathrm{o}} + \hat{a}_{\mathrm{o}}^{\dagger}\right)\left(\hat{p}_{\mathrm{k}}^{\dagger} + \hat{q}_{\mathrm{k}}^{\dagger} + \hat{p}_{\mathrm{k}} + \hat{q}_{\mathrm{k}}\right) \\ & + \sum_{\mathrm{k}} g_{\mathrm{i}}\left(\hat{a}_{\mathrm{i}} + \hat{a}_{\mathrm{i}}^{\dagger}\right)\left[e^{-i\varphi_1}\left(\hat{p}_{\mathrm{k}}^{\dagger} + \hat{q}_{\mathrm{k}}\right) + e^{i\varphi_1}\left(\hat{p}_{\mathrm{k}} + \hat{q}_{\mathrm{k}}^{\dagger}\right)\right] \\ & + \sum_{\mathrm{k}} g_{\mathrm{i}}\left(\hat{a}_{\mathrm{i}} + \hat{a}_{\mathrm{i}}^{\dagger}\right)\left[e^{-i(\varphi_1+\varphi_2)}\left(\hat{p}_{\mathrm{k}}^{\dagger} + \hat{q}_{\mathrm{k}}\right) + e^{i(\varphi_1+\varphi_2)}\left(\hat{p}_{\mathrm{k}} + \hat{q}_{\mathrm{k}}^{\dagger}\right)\right] \\ & + \sum_{\mathrm{k}} g_{\mathrm{o}}\left(\hat{a}_{\mathrm{o}} + \hat{a}_{\mathrm{o}}^{\dagger}\right)\left[e^{-i(\varphi_1+\varphi_2+\varphi_3)}\left(\hat{p}_{\mathrm{k}}^{\dagger} + \hat{q}_{\mathrm{k}}\right) + e^{i(\varphi_1+\varphi_2+\varphi_3)}\left(\hat{p}_{\mathrm{k}} + \hat{q}_{\mathrm{k}}^{\dagger}\right)\right], \end{aligned} \tag{S23}$$

where $\tilde{\omega}_{\mathrm{o(i)}} = \omega_{\mathrm{o(i)}} - i\beta_{\mathrm{o(i)}}$, and g_{i} (g_{o}) is the coupling strength between the inner (outer) GSE and the waveguide at the coupling point. As shown in Fig. 4, the propagating phases between the adjacent coupling points are denoted as φ_1, φ_2, and φ_3, respectively. Similar to the case of single GSE, the Heisenberg equation of the magnon modes can be obtained as

$$\begin{aligned} \frac{\mathrm{d}\hat{a}_{\mathrm{o}}}{\mathrm{dt}} = & - i\tilde{\omega}_{\mathrm{o}}\langle\hat{a}_{\mathrm{o}}\rangle - 2\kappa_{\mathrm{o}}\left[1 + \cos(\varphi_1+\varphi_2+\varphi_3) + \frac{i}{2}\sin(\varphi_1+\varphi_2+\varphi_3)\right]\langle\hat{a}_{\mathrm{o}}\rangle \\ & - (\Gamma + iJ)\langle\hat{a}_{\mathrm{i}}\rangle - i\sqrt{\kappa_{\mathrm{o}}}\left[1 + e^{i(\varphi_1+\varphi_2+\varphi_3)}\right]\hat{p}_{\mathrm{in}}, \end{aligned} \tag{S24}$$

$$\begin{aligned} \frac{\mathrm{d}\hat{a}_{\mathrm{i}}}{\mathrm{dt}} = & - i\tilde{\omega}_{\mathrm{i}}\langle\hat{a}_{\mathrm{i}}\rangle - 2\kappa_{\mathrm{i}}\left[1 + \cos\varphi_2 + \frac{i}{2}\sin\varphi_2\right]\langle\hat{a}_{\mathrm{i}}\rangle - (\Gamma + iJ)\langle\hat{a}_{\mathrm{o}}\rangle \\ & - i\sqrt{\kappa_{\mathrm{i}}}\left[e^{i\varphi_1} + e^{i(\varphi_1+\varphi_2)}\right]\hat{p}_{\mathrm{in}}, \end{aligned} \tag{S25}$$

where the dissipative coupling strength is

$$\Gamma = \sqrt{\kappa_{\mathrm{o}}\kappa_{\mathrm{i}}}\left[\cos\varphi_1 + \cos\varphi_3 + \cos(\varphi_1+\varphi_2) + \cos(\varphi_2+\varphi_3)\right], \tag{S26}$$

and the coherent coupling strength is

$$J = \frac{\sqrt{\kappa_{\mathrm{o}}\kappa_{\mathrm{i}}}}{2}\left[\sin\varphi_1 + \sin\varphi_3 + \sin(\varphi_1+\varphi_2) + \sin(\varphi_2+\varphi_3)\right]. \tag{S27}$$

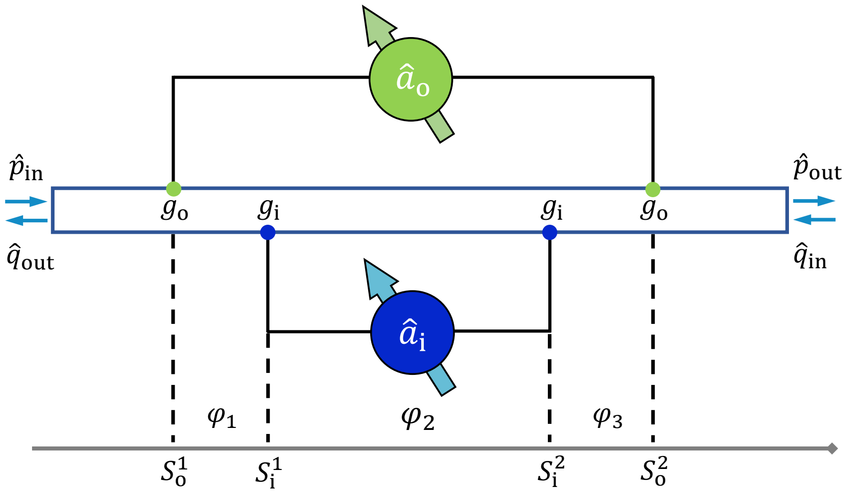

Supplementary Fig. 4 **Sketch of the nested two giant spin ensembles.**

Thus, the effective coupling strength between the two GSEs is given by $J - i\Gamma$.

The input-output relation for the nested configuration is

$$\hat{p}_{\rm out} = \hat{p}_{\rm in} - i\left[\sqrt{\kappa_{\rm o}}\left(1 + e^{-i(\varphi_1+\varphi_2+\varphi_3)}\right)\langle\hat{a}_{\rm o}\rangle + \sqrt{\kappa_{\rm i}}\left(e^{-i\varphi_1} + e^{-i(\varphi_1+\varphi_2)}\right)\langle\hat{a}_{\rm i}\rangle\right]. \tag{S28}$$

Owing to our symmetric design, the propagating phases φ_1 and φ_3 are equal. To fit the experiment data, we define the effective size of the outer (inner) GSE as $L_{\rm o,(i)} = S^2_{\rm o,(i)} - S^1_{\rm o,(i)}$, and the propagating phase is $\varphi = \omega L/v$. Using Eqs. (S24), (S25), and (S28), the transmission coefficient of the system can be solved as

$$\begin{aligned} S_{21}(\omega) =& 1 - i\left[\sqrt{\kappa_{\rm o}}\left(1 + e^{-i\frac{\omega L_{\rm o}}{v}}\right)\ \sqrt{\kappa_{\rm i}}\left(e^{-i\frac{\omega(L_{\rm o}-L_{\rm i})}{2v}} + e^{-i\frac{\omega(L_{\rm o}+L_{\rm i})}{2v}}\right)\right]\begin{bmatrix}\omega - \tilde{\omega}'_{\rm o} & i\Gamma - J\\ i\Gamma - J & \omega - \tilde{\omega}'_{\rm i}\end{bmatrix}^{-1} \\ &\times\begin{bmatrix}\sqrt{\kappa_{\rm o}}\left(1 + e^{i\frac{\omega L_{\rm o}}{v}}\right)\\ \sqrt{\kappa_{\rm i}}\left(e^{i\frac{\omega(L_{\rm o}-L_{\rm i})}{2v}} + e^{i\frac{\omega(L_{\rm o}+L_{\rm i})}{2v}}\right)\end{bmatrix}, \end{aligned} \tag{S29}$$

where

$$\tilde{\omega}'_{\rm o} = \omega_{\rm o} - \kappa_{\rm o}\sin\frac{\omega_{\rm o}L_{\rm o}}{v} - i\left[2\kappa_{\rm o}\left(1 + \cos\frac{\omega_{\rm o}L_{\rm o}}{v}\right) + \beta_{\rm o}\right], \tag{S30}$$

and

$$\tilde{\omega}'_{\rm i} = \omega_{\rm i} - \kappa_{\rm i}\sin\frac{\omega_{\rm i}L_{\rm i}}{v} - i\left[2\kappa_{\rm i}\left(1 + \cos\frac{\omega_{\rm i}L_{\rm i}}{v}\right) + \beta_{\rm i}\right] \tag{S31}$$

are the complex frequencies of the two GSEs. We plot transmission mapping versus the frequency detuning between the two GSEs using the parameters in Table I. The theoretical results agree well with the experimental data, as shown in Fig. 4 of the main text.

To clearly see the frequency-dependent radiative decay and Lamb shift of the GSE, it is useful to have an analytical solution for the integral

$$I(t,\tau,t_{12}) = \int_0^\infty J(\omega)d\omega\int_0^{t_{\rm f}} d\tau e^{i\omega(t-\tau-t_{12})}\left(\hat{a}^\dagger_{\rm m}(\tau) + \hat{a}_{\rm m}(\tau)\right). \tag{S32}$$

We use the approximation

$$\hat{a}_{\rm m}(\tau) \approx e^{-i\omega_{\rm m}(\tau-t)}\hat{a}_{\rm m}(t). \tag{S33}$$

Then, we let $x = \omega_{\rm m}(t-\tau)$, and the integral in Eq. (S32) can be written as

$$I(t,\tau,t_{12}) = \int_0^\infty J(\omega)d\omega\int_0^{\omega_{\rm m}t_{\rm f}}\frac{e^{-i\omega t_{12}}}{\omega_{\rm m}}\left[\hat{a}_{\rm m}e^{-i\frac{(\omega-\omega_{\rm m})x}{\omega_{\rm m}}} + \hat{a}^\dagger_{\rm m}e^{-i\frac{(\omega+\omega_{\rm m})x}{\omega_{\rm m}}}\right]dx. \tag{S34}$$

Mathematically, the integration over x is an oscillating function. If $t_{\rm f} \gg 1/\omega_{\rm m}$, the integration is over many periods, and the upper limit $\omega_{\rm m}t_{\rm f}$ of the integral can be regarded as an infinity. This approximation is feasible in waveguide magnonics due to the fact that the magnon frequency is on the order of several gigahertz, and the interaction time is determined by the decay rate, which is on the order of microseconds. For a 1D transmission line, the density of states can be generally regarded as "Ohmic" [4]. By using Eq. (S19), the integral in Eq. (S34) can be calculated as

$$\begin{aligned} I(t,\tau,t_{12}) &= \int_0^\infty d\omega\int_0^\infty\frac{\omega e^{-i\omega t_{12}}}{\omega_{\rm m}}\left[\hat{a}_{\rm m}e^{-i\frac{(\omega-\omega_{\rm m})x}{\omega_{\rm m}}} + \hat{a}^\dagger_{\rm m}e^{-i\frac{(\omega+\omega_{\rm m})x}{\omega_{\rm m}}}\right]dx \\ &= \int_0^\infty d\omega\frac{\omega e^{-i\omega t_{12}}}{\omega_{\rm m}}\left[\left(\pi\delta(\omega-\omega_{\rm m}) - i{\rm PV}\left(\frac{\omega_{\rm m}}{\omega-\omega_{\rm m}}\right)\right)\hat{a}_{\rm m} + \left(\pi\delta(\omega+\omega_{\rm m}) - i{\rm PV}\left(\frac{\omega_{\rm m}}{\omega+\omega_{\rm m}}\right)\right)\hat{a}^\dagger_{\rm m}\right]dx \\ &= e^{-i\omega t_{12}}\pi\left(\hat{a}_{\rm m} + \hat{a}^\dagger_{\rm m}\right) - i{\rm PV}\left\{\int_0^\infty e^{-i\omega t_{12}}\left[\left(\frac{\omega}{\omega-\omega_{\rm m}}\right)\hat{a}_{\rm m} + \left(\frac{\omega}{\omega+\omega_{\rm m}}\right)\hat{a}^\dagger_{\rm m}\right]d\omega\right\} \\ &= e^{-i\omega t_{12}}\pi\left(\hat{a}_{\rm m} + \hat{a}^\dagger_{\rm m}\right) - i(A - iB)\left(\hat{a}_{\rm m} + \hat{a}^\dagger_{\rm m}\right). \end{aligned} \tag{S35}$$

Then, we solve the imaginary and real parts of the integral coefficients of $\hat{a}_{\rm m}$. We define $x=(\omega\pm\omega_{\rm m})/\omega_{\rm m}$, $y=x\mp 1$, and add the converging factor $e^{-\alpha y}$. Thus, we have

$$\begin{aligned}
A &= \mathrm{PV}\left[\int_0^\infty \frac{\omega\cos(\omega t_{12})}{\omega\pm\omega_{\rm m}}d\omega\right] \qquad \text{(S36)}\\
&= \int_0^\infty \cos(\omega_{\rm m}t_{12}y)\,dy \mp \mathrm{PV}\left\{\int_{\pm1}^\infty \frac{\cos[\omega_{\rm m}t_{12}(x\mp1)]}{x}dx\right\}\\
&= \lim_{\alpha\to0^+}\int_0^\infty \cos(\omega_{\rm m}t_{12}y)\,e^{-\alpha y}dy \mp \left\{\cos(\omega_{\rm m}t_{12})\,\mathrm{PV}\left[\int_{\pm1}^\infty \frac{\cos(\omega_{\rm m}t_{12}x)}{x}dx\right] \pm \sin(\omega_{\rm m}t_{12})\,\mathrm{PV}\left[\int_{\pm1}^\infty \frac{\sin(\omega_{\rm m}t_{12}x)}{x}dx\right]\right\}\\
&= \lim_{\alpha\to0^+}\frac{\alpha}{(\omega_{\rm m}t_{12})^2+\alpha^2} \mp \left[-\cos(\omega_{\rm m}t_{12})\,Ci(|\omega_{\rm m}t_{12}|) + \frac{\sin(\omega_{\rm m}t_{12})}{2}\left[\pm\pi sgn(\omega_{\rm m}t_{12}) - 2Si(\omega_{\rm m}t_{12})\right]\right]\\
&= \pi\left[\mp M(\omega_{\rm m}t_{12}) + \sin(\omega_{\rm m}t_{12})\frac{\pm1-1}{2}\right],
\end{aligned}$$

and

$$\begin{aligned}
B &= \mathrm{PV}\left[\int_0^\infty \frac{\omega\sin(\omega t_{12})}{\omega\pm\omega_{\rm m}}d\omega\right] \qquad \text{(S37)}\\
&= \int_0^\infty \sin(\omega_{\rm m}t_{12}y)\,dy \mp \mathrm{PV}\left\{\int_{\pm1}^\infty \frac{\sin[\omega_{\rm m}t_{12}(x\mp1)]}{x}dx\right\}\\
&= \lim_{\alpha\to0^+}\int_0^\infty \sin(\omega_{\rm m}t_{12}y)\,e^{-\alpha y}dy \mp \left\{\sin(\omega_{\rm m}t_{12})\,\mathrm{PV}\left[\int_{\pm1}^\infty \frac{\cos(\omega_{\rm m}t_{12}x)}{x}dx\right] \mp \sin(\omega_{\rm m}t_{12})\,\mathrm{PV}\left[\int_{\pm1}^\infty \frac{\sin(\omega_{\rm m}t_{12}x)}{x}dx\right]\right\}\\
&= \lim_{\alpha\to0^+}\frac{\omega_{\rm m}t_{12}}{(\omega_{\rm m}t_{12})^2+\alpha^2} \mp \left[\frac{\cos(\omega_{\rm m}t_{12})}{2}\left[\pi sgn(\omega_{\rm m}t_{12}) - 2Si(\omega_{\rm m}t_{12})\right] \pm \sin(\omega_{\rm m}t_{12})\,Ci(|\omega_{\rm m}t_{12}|)\right]\\
&= \pi\left[\mp N(\omega_{\rm m}t_{12}) + \frac{1}{\omega_{\rm m}t_{12}}\right],
\end{aligned}$$

where $M(x)$ and $N(x)$ are infinite as x approaches 0, but are 0 as x approaches 1. Then, we calculate the mean value, and deduct the renormalized electrostatic energy contribution based on the original calculation done by Bethe [5]. Finally, we obtain

$$\begin{aligned}
I(t,\tau,t_{12}) &= 2\pi e^{-i\omega_{\rm m}t_{12}}\langle\hat{a}\rangle + i\pi\sin(\omega_{\rm m}t_{12})\langle\hat{a}\rangle, \qquad \text{(S38)}\\
&= 2\pi\cos(\omega_{\rm m}t_{12})\langle\hat{a}\rangle - i\pi\sin(\omega_{\rm m}t_{12})\langle\hat{a}\rangle,
\end{aligned}$$

where the two terms in Eq. (S38) correspond to the radiative decay and Lamb shift, respectively. This is consistent with our expectations, as the relaxation rate and the Lamb shift are related via a Hilbert transform according to the Kramers-Kronig relation [6]. For the inner GSE with two separated coupling points, we can define the characteristic time $t_{12}=(S_{\rm i}^2-S_{\rm i}^1)/v$, which represents the traveling time between the two coupling points.

IV. Spectra fitting of the nested two giant spin ensembles

The transmission spectra plotted in Figs 2**c**-2**e** of the main text are fitted using Eq. (S21). The fitting parameters for the magnon mode and the travelling photon mode in the waveguide are outlined in Table I. The meandering waveguide is fabricated on a 0.813 mm thick RO4003C substrate, and the effective size of the inner and outer GSEs are $L_{\rm i}=8.28$ cm and $L_{\rm o}=16.56$ cm, respectively.

The fitted radiative rates at various frequencies are depicted in Fig. 3**a** of the main text, which agree well with Eq. (S22). The non-radiative rates of two GSEs are estimated to be $\beta_{\rm i}/2\pi=1.581$ MHz and $\beta_{\rm o}/2\pi=1.391$ MHz, respectively. The transmission spectrum of the nested two giant spin ensembles can be fitted by

$$S_{21}(\omega)=1-\frac{2i\sqrt{\kappa_{\rm i,G}\kappa_{\rm o,G}}\,(J-i\Gamma)+i\kappa_{\rm i,G}\left[\omega-\omega_{\rm o}+i(\kappa_{\rm o,G}+\beta_{\rm o})\right]+i\kappa_{\rm o,G}\left[\omega-\omega_{\rm i}+i(\kappa_{\rm i,G}+\beta_{\rm i})\right]}{\left[\omega-\omega_{\rm o}+i(\kappa_{\rm o,G}+\beta_{\rm o})\right]\left[\omega-\omega_{\rm i}+i(\kappa_{\rm i,G}+\beta_{\rm i})\right]-(i\Gamma-J)^2}. \qquad \text{(S39)}$$

Using Eq. (S39), we can determine the coherent and dissipative coupling strengths between the two GSEs. The fitted coupling strengths are consistent with the theoretical predictions based on Eqs. (S26) and (S27). Two complete sets

Supplementary Table I Parameters used for fitting the single giant spin ensemble

Type	Bare radiative decay rate $\kappa_{\rm i}, \kappa_{\rm o}/2\pi$ (MHz)	Intrinisic decay rate $\beta_{\rm i}, \beta_{\rm o}/2\pi$ (MHz)	Effective size $L_{\rm i}, L_{\rm o}$ (cm)	Microwave speed v (m/s)
Inner GSE	0.76	1.58	16.56	3.26×10^7
Outer GSE	0.70	1.39	8.28	

Supplementary Table II Parameters used for fitting the nested two giant spin ensembles

$\omega_{\rm m}$ (GHz)	$\kappa_{\rm i,G}/2\pi$ (MHz)	$\kappa_{\rm o,G}/2\pi$ (MHz)	$\kappa_{\rm i,T}/2\pi$ (MHz)	$\kappa_{\rm o,T}/2\pi$ (MHz)	$\Gamma/2\pi$ (MHz)	$J/2\pi$ (MHz)
4.35	1.15	1.26×10^{-4}	2.69	0.86	3.28×10^{-4}	1.01
4.96	2.98	2.78	4.82	4.06	2.89	6.11×10^{-4}

of fitting parameters at the two concerned resonance frequencies (4.35 GHz and 4.96 GHz) are listed in Table II. The fitting curves are displayed in Figs. 4**d,h** of the main text.

* Electronic address: yipuwang@zju.edu.cn
† Electronic address: jqyou@zju.edu.cn

[1] Macdonald, J. Ferromagnetic resonance and the internal field in ferromagnetic materials. *Proceedings of the Physical Society, Section A* **64**, 968 (1951).
[2] Holstein, T. & Primakoff, H. Field dependence of the intrinsic domain magnetization of a ferromagnet. *Physical Review* **58**, 1098 (1940).
[3] Lalumiere, K. *et al.* Input-output theory for waveguide qed with an ensemble of inhomogeneous atoms. *Physical Review A* **88**, 043806 (2013).
[4] Kockum, A. F., Delsing, P. & Johansson, G. Designing frequency-dependent relaxation rates and lamb shifts for a giant artificial atom. *Physical Review A* **90**, 013837 (2014).
[5] Bethe, H. A. The electromagnetic shift of energy levels. *Physical Review* **72**, 339 (1947).
[6] Tannoudji, C. C., Grynberg, G. & Dupont-Roe, J. Atom-photon interactions (1992).
